# Can We Translate Vitamin D Immunomodulating Effect on Innate and Adaptive Immunity to Vaccine Response?

**DOI:** 10.3390/nu7032044

**Published:** 2015-03-20

**Authors:** Pierre Olivier Lang, Richard Aspinall

**Affiliations:** 1 Geriatric medicine and Geriatric rehabilitation division, Department of medicine, University Hospital of Lausanne (CHUV), CH-1011 Lausanne, Switzerland; 2 Health and Wellbeing academy, Anglia Ruskin University, CM1 1SQ Cambridge, UK; E-Mail: pierre-olivier.lang@chuv.ch

**Keywords:** vitamin D, vaccine, immunogenicity, immune cells, adaptive immunity, innate immunity, cholecalciferol, calcitriol, 25(OH)VitD

## Abstract

Vitamin D (VitD), which is well known for its classic role in the maintenance of bone mineral density, has now become increasingly studied for its extra-skeletal roles. It has an important influence on the body’s immune system and modulates both innate and adaptive immunity and regulates the inflammatory cascade. In this review our aim was to describe how VitD might influence immune responsiveness and its potential modulating role in vaccine immunogenicity. In the first instance, we consider the literature that may provide molecular and genetic support to the idea that VitD status may be related to innate and/or adaptive immune response with a particular focus on vaccine immunogenicity and then discuss observational studies and controlled trials of VitD supplementation conducted in humans. Finally, we conclude with some knowledge gaps surrounding VitD and vaccine response, and that it is still premature to recommend “booster” of VitD at vaccination time to enhance vaccine response.

## 1. Introduction

Vitamin D (VitD) is fat-soluble vitamin, unique in that it is primarily derived from the action of UV-B on the epidermis (VitD_3_ only) rather than absorbed from the diet (VitD_2_ and D_3_). Only plants and fungi synthetize VitD_2_ while VitD_3_ is physiologically produced in relatively large quantities in humans and the majority of vertebrates [1]. Because, worldwide, naturally occurring dietary sources of VitD are very limited, and food fortification is often suboptional, inconsistent, inadequate, or non-existent, individuals mainly depend on sunlight exposure for most of their requirements [2]. Of course, both type of VitD (D_2_ and D_3_) supplements are available for over-the-counter purchase; VitD_3_ is the type that most experts believe should be utilized in practice. Indeed, VitD_3_ is more effective at raising and maintaining the 25(OH)VitD serum level. Compared to D_3_, VitD_2_ does not bind as well to the VitD receptor (VDR) in human tissues. VitD_3_ is also more stable on the shelf compared to D_2_ as it is more likely to remain active for a longer period of time and when it is exposed to different conditions (*i.e.*, temperature, humidity, and storage). This has contributed to VitD_3_ being the most utilized form of VitD in clinical trials [3].

Through the action of UV-B, VitD is metabolized from 7-dehydroxycholesterol in the epidermis and converted at first in the liver to form its main circulating form. 25(OH)VitD which is then transported into the blood stream to the proximal tubule of the kidney where it is 1-α hydrolysed to form 1,25(OH)_2_VitD by the cytochrome P450 27B1 (*i.e.*, CYP27B1 or 25-hydroxyvitamin D 1-α hydroxylase) [4]. Once activated, VitD interacts with its specific receptor (*i.e.*, the VDR) in order to regulate expression and transcription of many genes and down stream products. This specific receptor, which is also known as NR1I1 (nuclear receptor subfamily 1, group I, member 1), is localized in the nucleus of various cell types [5,6]. Upon activation, the VDR forms a heterodimer with the retinoid-X receptor and binds to hormone response elements on DNA resulting in expression or transrepression of specific gene products.

The beneficial effects of VitD on skeletal homeostasis are very well documented, however the optimal serum 25(OH)VitD level at which these effects are obtained is still hotly debated among researchers in the world of VitD [7,8,9]. It is generally recommended to maintain the serum level above 75 nmol/L (*i.e.*, 30 ng/mL) [8], which is the VitD serum concentration associated with maximal parathyroid hormone (PTH) suppression [9,10]. Consequently, a serum concentration <50 nmol/L (*i.e.*, <20 ng/L) is considered as deficient and between 50 to 75 nmol/L (*i.e.*, 20 to 32 ng/L) as insufficient [11]. These current target serum concentrations were however determined for its application in bone health, but it is now becoming more and more clear that the tissue levels of VitD are dissociated from the serum levels that control VitD effects. The recent dramatic increase of data highlighting the extra-skeletal benefits of VitD and poor health outcomes associated with impaired VitD status has further stimulated the debate around the serum threshold for maximum health [7]. Indeed, the exact serum level that is associated with full tissue saturation is not known yet and consequently, the effective dose eliciting an effect on the immune system *in vivo*, for example, remains to be determined [10,12]. This question is also relevant for the role of VitD in inducting differentiation and inhibiting proliferation of various normal and cancer cells by regulating gene expression in multiple signaling pathways in a large number of tissues [12,13,14].

Recently, the potential role of VitD in modulating host defense to foreign antigens and pathogens has been particularly reported. This arises from the discovery that: (*i*) the immune system cells are able to produce CYP27B1 and to convert 25(OH)VitD into 1,25(OH)_2_VitD; (*ii*) the majority of cells composing both arms of the immune system (*i.e.*, innate and adaptive immunity) express VDR, mainly after they themselves have been stimulated; (*iii*) impaired VitD status which is common in many populations across the globe contributes to the burden associated with different pathogen infections; and (*iv*) the current body of evidence supports the view that VitD supplementation holds promise as a risk-modifying intervention in some infectious diseases [7,14,15,16].

The aim of this review is to describe how VitD regulates the immune response and its potential immumodulating roles with a specific focus on vaccine immunogenicity. Thus, we first consider the literature providing molecular and genetic supports to the idea that VitD status modulates innate and adaptive immune responses and hence vaccine immunogenicity. Then we discuss observational and control studies of VitD supplementation on vaccine response and effectiveness in human. Finally we conclude with some of the knowledge gaps surrounding VitD and vaccine response that should be further addressedl.

## 2. The Immune System: The Art of War

The immune system is crucial for human health and survival. With a suboptimally functioning immune system, even minor pathogens may prove fatal. Successful immunity requires a network of cells and effector molecules working together against invading foreign antigens in the daily battle with microorganisms and pathogens. Those cellular components are bone marrow derived B-cells and thymus derived T-lymphocytes that form part of the adaptive immune system; and macrophages, dendritic cells (DCs), granulocytes, and natural killer (NK) cells which compose the innate arm. In response to unknown or foreign antigens whatever their origins (e.g., pathogens, cancers and/or vaccines), a number of cytokines are produced by the cellular component among which are interleukin (IL) 1, IL-6, tumor necrosis factor α (TNFα), and interferon γ (INFγ). Normally, innate immunity cells first sense antigens through their toll like receptors (TLR), which once activated, induce the production of soluble mediators that are known to have systemic effects. Phagocytes (*i.e.*, macrophages, neutrophils, NK cells), with their ability to engulf foreign particles, are central to the innate immune response, which is a nonspecific immune response. Once activated, the innate immune response calls upon T- and B-lymphocytes that increase the power and focus of the immune response. This contribution to defense is called the adaptive immune response because it is organized around ongoing attack and adapted to the nuances of foreign antigens. Consequently, adaptive immunity provides a highly specialized and specific system of defense against one particular antigen. Conversely this is of little effect against an attack associated with different antigen. This explains, for example, why optimal protection from influenza vaccine is so dependent on the exact matching between circulating influenza strains and the antigens contained in the vaccine. The most important consequence of an adaptive immune response is the establishment of a state of immunological memory (called primary response). Immunological memory is the ability of the immune system to respond more rapidly and effectively to pathogens that have been encountered previously. It reflects the preexistence of a clonally expanded population of antigen-specific lymphocytes, which persist in the body. Memory responses, which are called secondary, tertiary, and so on, depending on the number of exposures to antigen, also differ qualitatively from primary responses. This is particularly clear in the case of the antibody response, where the characteristics of antibodies produced in secondary and subsequent responses are distinct from those produced in the primary response to the same antigen. Memory T-cell responses have been harder to study, but can also be distinguished from the responses of naive or effector T-cells [17]. This long-lasting memory response is the aim of vaccination and immunization programs at individual and population levels respectively [18].

## 3. Immunomodulating Role of VitD in Host Protection

**Figure 1:**
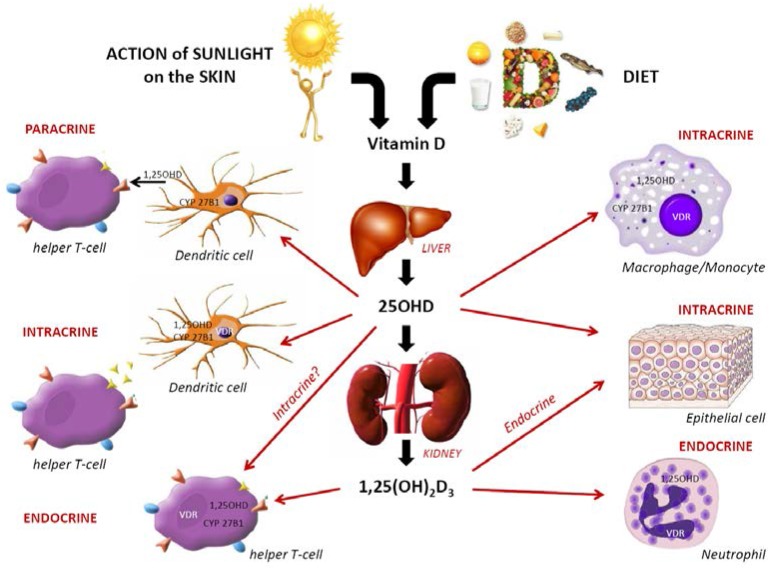

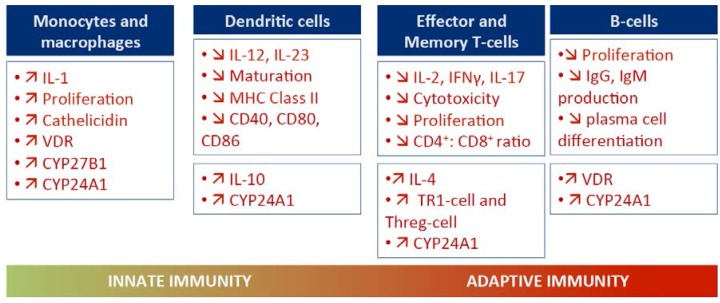
Mechanisms by which 25(OH)VitD and 1,25(OH)_2_VitD modulate innate and adaptive immune response (up) and overview of immunomodulatory actions on monocytes and macrophage; dendritic cells; and effector and memory T and B-cells (down). All these cells possess the enzyme (CYP27bB1) for hydroxylation steps to generate 1,25(OH)_2_VitD. Through endocrine, intracrine and paracrine mechanisms, the active form of VitD binds to the VitD receptor (VDR) to induce a wide range of immunological effects (adapted from Lang *et al.* [7]).

The finding that the majority of immune cells can express VDR and part of them produce CYP27B1, has raised the idea that VitD could have pleiotropic effects on human immunity [12,14,19]. As depicted in Figure 1, it is now clear that VitD has effects on both arms of the immune system and moreover those effects are not limited to the classic endocrinal pathway of 1,25(OH)_2_VitD. Indeed, in addition it is also important to consider intracrine (*i.e.*, VitD acts inside a cell) and paracrine signaling pathways (*i.e.*, cell-cell communication in which a cell produces a signal to induce changes in nearby cells, modulating the behaviour or differentiation of those cells) that are subject to distinct set of modulatory signals [7,20]. Through those pathways, VitD is able to modulate innate and adaptive immunity as an immune switch hard to turn on, but easy to turn off. Indeed, VitD improves appropriate immune system communication by making it harder for foreign antigen to trigger an inflammatory response and also makes it easier to turn off the inflammatory cascade once it is initiated. It is, however, very important to note that most of the data presented below results from laboratory based *in vitro* studies in which exogenous 1,25(OH)_2_VitD was added at levels usually above physiological levels [7]. This therefore raises the question of whether VitD effectively interferes at physiological concentrations with immune cells and also suggests that intracellular synthesis of 1,25(OH)_2_VitD and subsequent intracrine activity could be more effective in achieving these tasks [7].

### 3.1. VitD and Innate Immunity

As depicted by the upper part of Figure 1, target cells such as monocytes/macrophages and DCs directly utilize circulating 25(OH)VitD for intracrine activity. In addition to phagocytosis of the foreign antigen, these cells also sense antigens through their TLRs which once activated up regulate the expression of genes that code for the VDR and CYP27B1 [21]. In DCs, the intracrine activity of VitD inhibits maturation and thereby modulates CD4_+_ T-cell function. The helper T-cell (Th) response is also modulated in a paracrine fashion, with 1,25(OH)_2_VitD acting on VDR-expressing CD4_+_ T-cells. Neutrophils, which do not express CYP27B1, are likely to be affected by circulating levels of the active form of VitD synthesized by the kidneys. VDR-expressing CD4^+^ T-cells are also potential targets for systemic 1,25(OH)_2_VitD [20]. The lower part of the Figure 1 specifically shows an overview of how 25(OH)VitD and 1,25(OH)_2_VitD modulate monocytes and macrophages, and DCs activation in terms of VDR, CYP27B1, CYP24A1 (*i.e.*, the enzyme degrading 1,25(OH)_2_VitD), production of soluble mediators, proliferation and maturation, and the expression of cell membrane receptors and through endocrine, paracrine, and intracrine mechanisms [14,22]. The active form of VitD also inhibits TLRs expression so inducing a state of hyporesponsiveness to antigen-induced molecular cascades. This could be considered as a negative feedback mechanism self-limiting excessive TLR activation and inflammation [23]. In addition, 1,25(OH)_2_VitD enhances antibacterial effect by interacting with the promoter of cathelicidin (hCAP) [15]. It so promotes microbial killing in phagocytic vacuoles and chemioattraction for neutrophils and monocytes [24]. However, in order to activate hCAP and enhance macrophage function, it is necessary to reach sufficient levels of circulating 25(OH)VitD [25]. Similarly, epithelial cells, trophoblasts and decidual cells are able to respond to an intracrine hydroxylation of 25(OH)VitD to promote antibacterial responses but they can also respond to systemic 1,25(OH)_2_VitD [26]. Systemic 12,25(OH)_2_VitD may also induce immune tolerance by down-regulating pro-inflammatory cytokines in myeloid DCs, such as the cluster of differentiation (CD)-40, CD-80, CD-86, major histocompatibility complex (MHC) class II, CD-54, IL-12, IL-23p40 and the C-C motif chemokine ligand (CCL) 17 [27].

### 3.2. VitD and Adaptive Immunity

The tissue-specific synthesis of calcitriol from circulating 25(OH)VitD has been shown to be important for both T-cells and B-cells immune response. As presented in Figure 1, once activated, DCs induces intracellular activation of 25(OH)VitD, which by intracrine activity inhibits DCs maturation. Thereby 1,25(OH)_2_VitD through intracrine and paracrine actions permits induction and modulation of an initial Th-response [27]. In this scenario, by acting as an inhibitor of maturation, 1,25(OH)_2_VitD inhibits T-cells proliferation [28]. Thus, the calcitriol signaling represses the transcription of genes encoding Th1 response-like cytokine (*i.e.*, INFγ which activates cell-mediated immunity—Macrophage, Cytotoxic T-cell, NK cells—and production of opsonizing antibodies by B-cells—to induce protection against intracellular pathogen) [29,30] and Th17 (*i.e.*, IL17 to provide anti-microbial immunity at epithelial and mucosal barriers) [31] in order to polarize the CD4^+^ T-cells responses toward a more regulatory Th2 (*i.e.*, IL2 which activate humoral-mediated immunity by the production of neutralizing antibodies by B-cells to induce protection against extracellular pathogen) [32] or Threg phenotypes [14,33]. These two last phenotypes are considered key components of VitD capacity to suppress Th1-driven autoimmunity responses [28,33]. Th17 cells also play a crucial role in combating certain pathogens (*i.e.*, *Candida albicans*, *Cryptococcus neoformans*, *Helicobacter pilory*, *Klebsiella pneumoniae*, *M. tuberculosis* and *Staphylococcus* [34]) but they have also been linked to tissue damage and inflammation [35,36]. Thus it seems that 1,25(OH)_2_VitD contributes to maintains self-tolerance by damping overly zealous adaptive immune system responses while enhancing protective innate responses [7]. However, the exact mechanisms by which variations in VitD status may influence T-cell functionalities are far to be fully elucidated [7].

Initially, it was though that the anti-proliferative effect of 1,25(OH)2VitD on B-cells was an indirectly mediated by CD4^+^ T-cells [14]. Recent *in vitro* analysis however observed that B-cells were capable of intracrine responses. Moreover, VDR expression in B-cells seems to also be regulated by 1,25(OH)_2_VitD suggested that the active form of VitD exerts differential effects on activated *versus* resting B-cells with probably different effects according to individuals’ levels of serum 1,25(OH)_2_VitD [37]. Indeed, the inhibitory effects mediated by the up-regulation of the VDR, as presented in Figure 1 (down), requires a certain level of VDR engagement to induce the anti-proliferative effect. Interestingly, CYP27B1 mRNA was also found expressed in resting B-cells. Inversely, CYP24A1 was found significantly up regulated following the incubation of human B cells with 1,25(OH)_2_VitD. This suggests that the activity of VitD might be influenced not only by VDR expression but also by the capacity to degrade the active molecule. In contrast to the VDR, CYP24A1 was not altered by B-cell activation, demonstrating that human B cells can respond to 1,25(OH)_2_VitD directly. The increased susceptibility of activated B cells to many of the effects of 1,25(OH)_2_D_3_ might reflect the up-regulation of VDR but not CYP24A1 by these cells [37]. 25(OH)VitD has similar effects on purified B-cells compared with the active form, but at higher concentrations [14]. Finally, 1,25(OH)_2_VitD limits ongoing B-cell proliferation and modulates B-cell responses. Its effects on plasma cell and memory cell differentiation however result from the suppression of ongoing B-cell proliferation [38], which is required before the differentiation steps can occur [39]. Other B-cells targets modulated by 1,25(OH)_2_VitD include IL-10 [40] and the chemokine receptor CCR10 [41], suggesting that B-cells responses to VitD are extended to regulating mucosal immunity [22,31].

### 3.3. Mechanisms by Which VitD May Induce Anti-Infection Protection

Recent studies, for the most part conducted in animal models and *ex vivo* human cells, provide insight into the involvement of VitD deficiency and its supplementation in the anti-microbial response to various bacterial (e.g., *Helicobacter pylori*, *Pseudomonas aeruginosa*, *Bordetella bronchoseptica*, *Salmonella,* and *Shigella*) and viral infections (e.g., *Influenzae*, *Respiratory Syncytial Virus*, *Human Immunodeficiency Virus*, and Hepatitis C virus) [7]. However, based on the ability of circulating 25(OH)VitD and 1,25(OH)_2_VitD to modulate innate and adaptive immune response (*i.e.*, suppression of the oxidative burst, Th1-mediated and B-cell immune responses), it would be expected that VitD impairs the host defenses to pathogens. Indeed, the Th1/Th17 immune responses are protective during infections [34] and 1,25(OH)_2_VitD would be predicted to increase Threg cells [42]. Moreover, analyses in VDR knock-out, wild-type and VitD deficient mice do not support either a beneficial or harmful effect of 1,25(OH)_2_VitD on host immunity with respect to a large range of pathogens (e.g., *Listeria monocytogenes*, *Leishamania major*, *Mycobacterium Bovis*, *Mycobacterium Tuberculosis*, *Candida Albicans*, *Herpes simplex*, *Shistosoma mansoni*, and *Bordetella pertussis*) that require either a Th1/Th17 or Th2-mediated immune response [7]. Finally, it appears that optimal VitD status has key immunomodulatory properties in infectious disease settings, by down-regulating excessive and therefore toxic cytokine responses while allowing the clearance of various pathogens through the production of antimicrobial peptides [14]. The capacity of the active form of VitD to regulate innate immunity and to induce production of hCAP or β*-*defensin [20] suggests a less straightforward outcome of any changes of 25(OH)VitD and/or 1,25(OH)_2_VitD. In monocytes/macrophages, the activation of TLRs induces expression of VDR and localized synthesis (*i.e.*, intracrine) of 1,25(OH)_2_VitD [20], that in turn, enhances hCAP synthesis [43]. Complementary studies have demonstrated that hCAP serves as a mediator of VitD-induced autophagy [31]. Finally effective innate immunity against many bacterial pathogens requires macrophage responses that up regulate phagocytosis and direct antimicrobial pathways [44]. It is also interesting to note that hCAP exhibits antiviral properties [45]. So it might enhance protection against viral diseases such as influenza [46] but without confirmation by *in vitro* or *in vivo* studies yet [15]. This VitD-associated induction of hCAP has been also reported in human cell types outside the immune system (e.g., keratinocytes, gastrointestinal and bronchial epithelial cells, myeloid cell lines, decidual and trophoblastic cells [7]]. Thus, some battles against pathogens at mucosal surfaces seem also to be regulated by VitD [31].

## 4. VitD Status and Vaccine Immunogenicity

### 4.1. Good Immune Response: The Key to Success of Vaccinations

**Figure 2:**
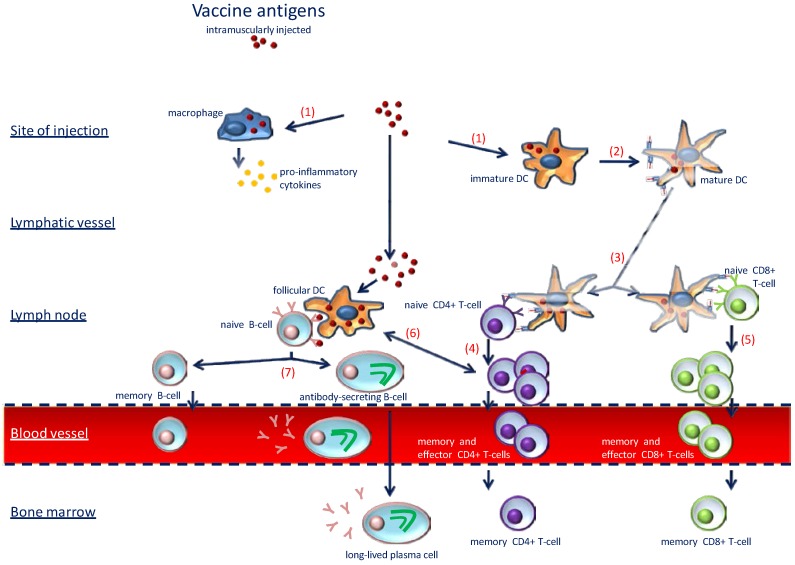
Schematic representation of the vaccine immune response. Administration of vaccine antigens induces the activation of the innate immune system. Antigens are first taken up by antigen-presenting cells (**1**). This local innate immune response facilitates maturation of dendritic cells (DCs) (**2**). Mature DCs migrate into lymph nodes (**3**), where they induce activation and clonal expansion of naive CD4^+^ (**4**) and CD8^+^ (**5**) T-cells. The activation and differentiation of naive B-cells is induced by antigen and CD4^+^ T helper cells (**6**) as well. Naive B-cells differentiate into memory B-cells and antibody-secreting B-cells (**7**). (Adapted from Lang *et al.* [47]).

Vaccines are powerful public health tools providing tremendous benefits in protecting vulnerable populations from life-threatening infectious diseases worldwide. Indeed, with the exception of safe water, no other modality, not even antibiotics, has had such a major positive impact on morbidity and mortality reduction [18]. With the introduction of several successful vaccines during the 20th century, major achievements included the development of the polio vaccine in the 1950s and the eradication of smallpox during the 1960s and 70s have been obtained. While the latter has contributed to the prevention of 300 million cases and probably saved 100 million lives, the incidence of poliomyelitis has been reduced by 100% and by 90% for diphtheria, tetanus, measles, mumps and rubella [47]. As depicted in Figure 2, immunization by vaccination stimulates an immune response through the activation of both innate and adaptive immunity. The administration of vaccine antigen (with or without adjuvant and whatever the route of administration) first induces the activation of innate immune response at the site of injection. Vaccine antigens are then captured, processed, and presented by antigen-presenting cells (APCs; *i.e.*, macrophages and DCs), which migrate into lymph nodes where they induce activation and clonal expansion of naïve CD4^+^ and CD8+ T-cells (helper and cytotoxic T-cells respectively). The subsequent activation and differentiation of naïve B-cells is induced by CD4+ T-cells, which differentiate into memory B-cells and antibody-secreting B-cells. Finally long-term immunity is maintained by memory B and T-cells (CD4+ and CD8+) in the blood stream and lymph nodes, as well as in the bone marrow by long-lived plasma cells and memory T-lymphocytes [47].

However, being vaccinated does not guarantee full protection from disease at all times. It is known and accepted that in younger infants and older adults vaccines are much less effective [48,49]. Although an individual’s age is a major contributor, there is no single cause of vaccine failure in the general population. Rather, there is a compilation of events, which include dysregulation of specific facets of human immunity and/or hormonal pathway, chronic inflammation, protein-energy malnutrition and/or micronutrients and vitamin deficiencies [47,48]. However, this list is far from a comprehensive understanding of the mechanisms and kinetics of the process that contributes to long-term memory and protection following vaccination.

The inability of the immune system to provide adequate protection from infection is however a feature of normal aging populations and the elderly more particularly. As a consequence, these populations demonstrate less effective response to vaccination because of the age-associated remodeling of the immune system [50]. Many studies have been carried out to find factors that contribute to the immunosenescence process. Results have identified causes related to DCs; T- and B-cell production, function as well as repertoires; and NK cells [47]. While the role played by nutritional status (*i.e.*, micro and macro-nutrition) and comorbidities has been investigated, whether the lower vaccine effectiveness of the elderly is related or not to VitD status, or whether VitD deficiency just further impinges a state of immune vulnerability, are still important but unsolved questions [4].

### 4.2. Translating Immunomodulatory Roles of VitD in Vaccine Response

With specific regard to vaccination the potential effects of VitD on vaccine response (see Figure 1) might be mediated via its action on APCs with the most potent reported effects on DCs [50,51]. Not only on activation via TLRs by vaccine antigen (with or without adjuvant) DCs up-regulate CYP27B1 [21] but by inducing T-cell proliferation and cytokine production. The latter results in inhibition of the Th1-like cytokines (IL-2, IFNγ), increase of the Th2-like cytokines (IL4, IL5, IL10 and IL13), and in production of Th17-like cytokine (IL17) secretion [52,53], and finally through indirect effects on B-cell homeostasis, proliferation and immunoglobulin production [37]. So although 1,25(OH)_2_VitD would seem to directly blunt B-cell functions (see Figure 1), it would, paradoxically, stimulate vaccine effectiveness through its effects on innate immunity [15].

Recent studies have confirmed the potent effects of VitD on cytokine production and in regulating normal innate and adaptive immune functions in animals and humans [15,54,55]. In animal models, it has been observed that locally produced 1,25(OH)_2_VitD induces migration of DCs from the site of vaccination to non-draining lymphoid organs, where they can stimulate antigen-specific T- and B-cells to mount a strong and persistent antibody response to diphtheria vaccination [56,57]. Co-administration of 1,25(OH)_2_VitD with trivalent influenza vaccine in mice enhances both mucosal and systemic specific antibody response, and subsequently the animals’ ability to neutralize live influenza virus instilled in the nose [58,59]. Other studies, also conducted in animal models or with *ex vivo* human cells have highlighted the role played by VitD deficiency and its supplementation on immune response to vaccination (e.g*.*, hepatitis B, measles, rubella, and *BCG* vaccinations) [60,61,62,63].

## 5. What Do We Know from Clinical Trial and Observational Studies?

Consecutively to the potent effects of VitD on cytokine production and regulation of immunity, the influence of its serum level and multiple VDR SNPs/haplotype on the vaccine immunogenicity and their potential roles as vaccine enhancer have been investigated in humans as summarized in Table 1. When 1.0 μg of 1,25(OH)_2_D_3_ was intramuscularly co-administered with influenza vaccine at the site adjacent to vaccination in a randomized control-study conducted in 175 healthy human volunteers (18–49 years of age), humoral immunity was not enhanced [64]. Serum influenza hemagglutination inhibition (HAI) titres were used to assess the ability of the host immune response to neutralize the infectivity of the virus [65]. However three important limitations are drawn from this study. First, the subjects were young and healthy and since serum 25(OH)VitD status was not measured at baseline, it is most probable that these individuals were neither VitD deficient nor insufficient. This reinforces the idea, as previously mentioned with respect to the host defense against pathogens, that higher than optimal levels (*i.e.*, 40 ng/mL or 100 nmol/L) do not seem to provide an additional benefit [15,66]. Second, significant pre-vaccination HAI titres were measured in nearly all subjects. This indicates that the subjects had considerable immunity to three vaccine influenza strains before their vaccination. Since a clear inverse relationship exists between pre-immunization serum HAI and antibody response after vaccination this could have masked the potential vaccine-enhancing effect of 1,25(OH)_2_VitD [15]. Third, VitD supplementation and vaccine were simultaneously administered; the delay between the two injections was too limited to expect any beneficial biological effects on DCs, on macrophages, or on cells composing the adaptive immunity. This is true even if the active form of VitD was considered in the study, because the endocrine activation pathway, as depicted in Figure 1 does not seem to be the main mechanism by which 1,25(OH)_2_VitD modulates innate immunity. More recently, the influence of VitD on influenza vaccine immunogenicity in HIV was assessed using data from a phase 3 randomized trial conducted during the 2008–2009 influenza season [67]. One third of participants were on supplemental VitD at baseline. Neither seroconversion nor seroprotection rates were predicted by VitD use for any of the 3 vaccine strains (A/H1N1, A/H3N2, and influenza B).

**Table 1:** Summary of clinical trials and observational studies evaluating the influence of VitD serum level and multiple VDR single nucleotide polymorphisms (SNP/haplotype) on the vaccine immunogenicity in humans.

Vaccines	Reference	Study		VitD Supplementation		VitD Status (post-Replacement)	Results
		Design	Population	Dosage (IU)	Duration		
Influenza	Kriesel *et al.* [64]	RCT	175 adults (USA)	40 **	One dose	**NR**	**A**
Hepatitis B	Moe *et al.* [68]	RCT	31 haemodialysis patients (USA)	144 μg *	12 weeks	**NR**	**A**
Herpes Zoster	Ginde *et al.* [69]	SGA	150 nursing home residents (60 years old or over—USA)	High dose *vs.* normal dose	4 months	**?**	**?**
				**VDR SNPs/Haplotype**			
Measles	Ovsyannikova *et al.* [61]	SGA	745 healthy children genotyped for the 391 polymorphisms in their VDR (11–22 years old—USA)	Association between multiple VDR SNPs/haplotype and vaccine adaptive immune response		**NR**	**C**
Rubella	Ovsyannikova *et al.* [62]	SGA	714 healthy children genotyped for the 148 candidate SNPs (11–19 years old—USA)	Association between polymorphism in VDR and vaccine adaptive immune response		**NR**	**C**
				**Yearly Season of Inoculation**			
Rubella	Linder *et al.* [70]	SGA	Children aged 4–5 years (Israel) divided in 3 groups according to the season of the year in which vaccination was performed	The association between vaccine immunogenicity and with sunlight variability-related to yearly season		**NR**	**C**

S = serum 25(OH)D_3_ significantly increased and/or corrected deficiency. The exact % of the population who achieved desirable levels is mentioned between brackets when informed; NS = No significant difference between the two groups; NR = Not reported; RCT: Randomized controlled trial; SGA: single group assignment; VitD: vitamin D; VDR: VitD receptor; SNPs: single nucleotide polymorphisms; IU: international unit; * paricalcitol = 19-nor-1,25(OH)_2_D_2_, being an analog of 1,25-dihydroxyergocalciferol, the active form of VitD_2_; ** intramuscularly administered; A = No study end-points met, NEGATIVE study; B = All study end-points met, POSITIVE study; C = Some study end-points met, MIXED study.

Similarly, in a randomized controlled trial (RCT) conducted in thirty-one haemodialysis patients with standard hepatitis B booster vaccine, the intravenous administration of 4 μg of paricalcitol (*i.e.*, an analog of the active form of VitD_2_) with the haemodialysis session three times weekly over 12 weeks were compared to placebo. The *in vivo* memory response and *in vitro* proliferation and release of IL-2, IL-6, TNF-α, and INF-γ were not different between the groups [68]. In contrast to clinical immune effects, paricalcitol increased serum calcium levels and decreased further PTH and bone alkaline phosphatase levels significantly. However, patients were not under VitD supplementation at baseline and both exact serum 25(OH)VitD at baseline and after complementation were not reported. Finally, one RCT is ongoing to determine the increase in the 3 weeks post-vaccination specific cell-mediated immune response from herpes zoster vaccination in 150 nursing home residents (60 years old or older) after 4 months of high dose *vs.* standard dose VitD_3_ supplementation [69] (final data collection and primary outcome measure: December 2014). Interestingly, specific allelic variations and haplotypes of the VDR genes seem to also modulate the specific immune response to vaccine. Using a tag SNP (*i.e.*, representative single nucleotide polymorphism) approach, 745 healthy children were genotyped for the 391 polymorphisms in their VDR genes [61]. Significant associations between multiple VDR SNPs/haplotypes and measles-specific IL-2, IL-6, IL-10, IFN-α, IFN-γ, IFNλ-1, and TNF-α cytokine secretions were found. This suggests that allelic variations and haplotypes in the VDR genes influence adaptive immune responses to measles vaccine. Similarly, Ovsyannikova *et al.* [62] genotyped 714 healthy children for 148 candidate SNP markers and presented evidence that, after two doses of rubella-vaccine, polymorphisms in VDR, but also in genes modulating innate immunity influence adaptive cytokine responses.

Finally, it has been analyzed whether the season of inoculation may modulate the immune response to vaccination. Indeed, the yearly seasons are marked by changes in the amount of sunlight and UV-radiation is known to adversely affect the course of viral infections, immunologic memory and cellular and humoral immune responses [3,7]. Linder *et al.* have thus investigated potential differences in the immune response to the rubella vaccine after 3–4 years by season of inoculation in children aged 4–5 years attending four kindergartens in villages in northern Israel [70]. All of them had been vaccinated at 1 year of age. Participants were divided into three groups according to the season of the year in which the vaccination was performed. Of the 203 children tested, 186 (91.6%) had adequate antibody levels, 7 (3.4%) had equivocal levels and 10 (4.9%) had inadequate levels. Significantly higher mean geometrical titers were found in the winter-inoculated compared with the summer-inoculated group. The same tendency was noted in the percent of infants properly immunized. Thus, this preliminary study suggests that booster vaccine immunogenicity may be improved by inoculating children during seasons of less sunlight or by reducing the children’s exposure to sunlight following inoculation. However, further studies are needed to corroborate and expand these controversial findings.

## 6. Conclusions

Indubitably, the current idea that 25(OH)VitD and 1,25(OH)_2_VitD are more than just key players in bone formation is acceptable. This review has clearly shown that the cellular component of both arms of the immune system are not only targets for the active form of VitD but are also able to activate circulating 25(OH)VitD arguing for intracrine and paracrine activities in addition to the classic endocrine pathway.

While the current understanding supports the view that VitD insufficiency/deficiency increases susceptibility to various pathogens [7], its potential impact on vaccine immunogenicity is far from being demonstrated. Thus the concept that VitD is better than any vaccine or could be an immune or vaccine booster is not proved yet. Indeed, the underlying mechanisms that may lead to immunogenicity enhancement (or vaccine hyporesponsiveness according to VitD status) are yet to be clarified and further investigated. Although evidence about skeletal benefits supports the need of maintaining 25(OH)VitD at the desirable level, our understanding of non-classical immunological actions of VitD is far from complete. Moreover, the current target serum concentration of 75 nmol/L and dosage recommendations for VitD revolves around its application in bone health, while the effective dose eliciting an effect of the immune system *in vivo* remains to be determined. Thus, there is not yet sufficient information to clarify the true relationship between VitD status and vaccine immunogenicity. Therefore, it is still premature to recommend “booster” VitD supplementation at vaccination time to enhance vaccine response.
